# Potential critical risks of pulmonary thromboembolism from an asymptomatic postpartum ovarian vein thrombosis: a case report

**DOI:** 10.1186/s12884-022-04627-w

**Published:** 2022-04-15

**Authors:** Tsuyoshi Murata, Yuki Yoshimoto, Yoshiaki Shibano, Koji Owada, Masayuki Miyajima, Soichi Nakamura, Ryuji Yamauchi

**Affiliations:** 1 Department of Obstetrics and Gynecology, Shirakawa Kosei General Hospital, 2-1 Toyochi Kamiyajiro, Shirakawa, Fukushima, 961-0005 Japan; 2 Department of Circulatory Internal Medicine, Shirakawa Kosei General Hospital, Fukushima, 961-0005 Japan; 3 Department of Radiology, Shirakawa Kosei General Hospital, Fukushima, 961-0005 Japan

**Keywords:** Ovarian vein thrombosis, Deep vein thrombosis, Pulmonary thromboembolism, Postpartum, Case report

## Abstract

**Background:**

Ovarian vein thrombosis (OVT) may cause maternal mortality by inducing pulmonary thromboembolism (PTE). However, the prevalence, etiology, risk factors, prognosis, and optimal treatments for asymptomatic OVT during and after pregnancies are unclear, which therefore requires a high clinical index of suspicion for certain diagnoses due to its vague presentation. We herein present a case of asymptomatic postpartum OVT that extended toward the inferior vena cava (IVC), resulting in a potential risk of PTE.

**Case presentation:**

A 30-year-old postpartum woman presented with slight dyspnea after an uneventful vaginal delivery at 40 weeks of gestation. We checked her laboratory data to exclude lethal thrombosis; D-dimer levels were 85.6 μg/mL. We performed computed tomography (CT) to search the presence of PTE and deep vein thrombosis (DVT); although no signs of PTE and DVT in her legs were detected, CT and trans-abdominal ultrasonography (TAUS) revealed a right OVT. Heparin was administered, and D-dimer levels decreased; warfarin at a dose of 2 mg/day was subsequently administered to control anti-coagulopathy. However, D-dimer was re-elevated despite adequate anticoagulation treatment, and extension of the right OVT to the IVC was detected by CT and TAUS. With warfarin administration, CT and TAUS showed the disappearance of right OVT. The patient was discharged from the hospital 17 days after delivery.

**Conclusions:**

Even asymptomatic postpartum OVT may lead to PTE. Universal screening guidelines and optimal treatment strategies for asymptomatic OVT in pregnant and postpartum women should be established through future studies.

## Background

Ovarian vein thrombosis (OVT) is a rare thrombotic condition with an incidence 60-fold lower than that of deep vein thrombosis (DVT) located in the legs [1]. It can occur in both pregnant and non-pregnant women; 1 per 500 to 1 per 2000 pregnancies and 0.18% of the general population were reported to show complications of OVT [1, 2]. Specifically, pregnancy-related OVT has a high rate of resolution after short-term treatment [3]. OVT during and after pregnancies often presents with fever and lower abdominal pain [4], which could occur after both vaginal and cesarean deliveries [5–7]. OVT is considered critical because of its potential risks for pulmonary thromboembolism (PTE) [5, 8].

Although symptomatic postpartum OVT complicates 0.01–0.05% of deliveries, asymptomatic postpartum OVT might be more common; one report indicated that signs of OVT were found in 30% of women post low-risk vaginal delivery on magnetic resonance venography [4, 9]. However, this high prevalence suggests that OVT has uncertain clinical significance [9]. Thus, the detailed prevalence, etiology, risk factors, prognosis, and optimal treatments for asymptomatic OVT during and after pregnancies are unclear [1], requiring a high clinical index of suspicion for certain diagnoses due to its vague presentation [3].

We herein present a case where asymptomatic postpartum OVT was incidentally found on computed tomography (CT), which showed that there is a potential critical risk where even asymptomatic OVT can lead to PTE based on the finding of OVT extending to the inferior vena cava (IVC) despite adequate anticoagulation treatment.

## Case presentation

A 30-year-old healthy, nulliparous pregnant woman exhibited an uneventful pregnancy course. She was admitted to the hospital due to onset of labor at 40 weeks of gestation. Labor was reinforced because of weak contractions. She delivered a healthy female newborn (weight: 3478 g; Apgar score at 1 and 5 min: 9 at both times) at 40 weeks and 3 days of gestation.

**Fig. 1:**
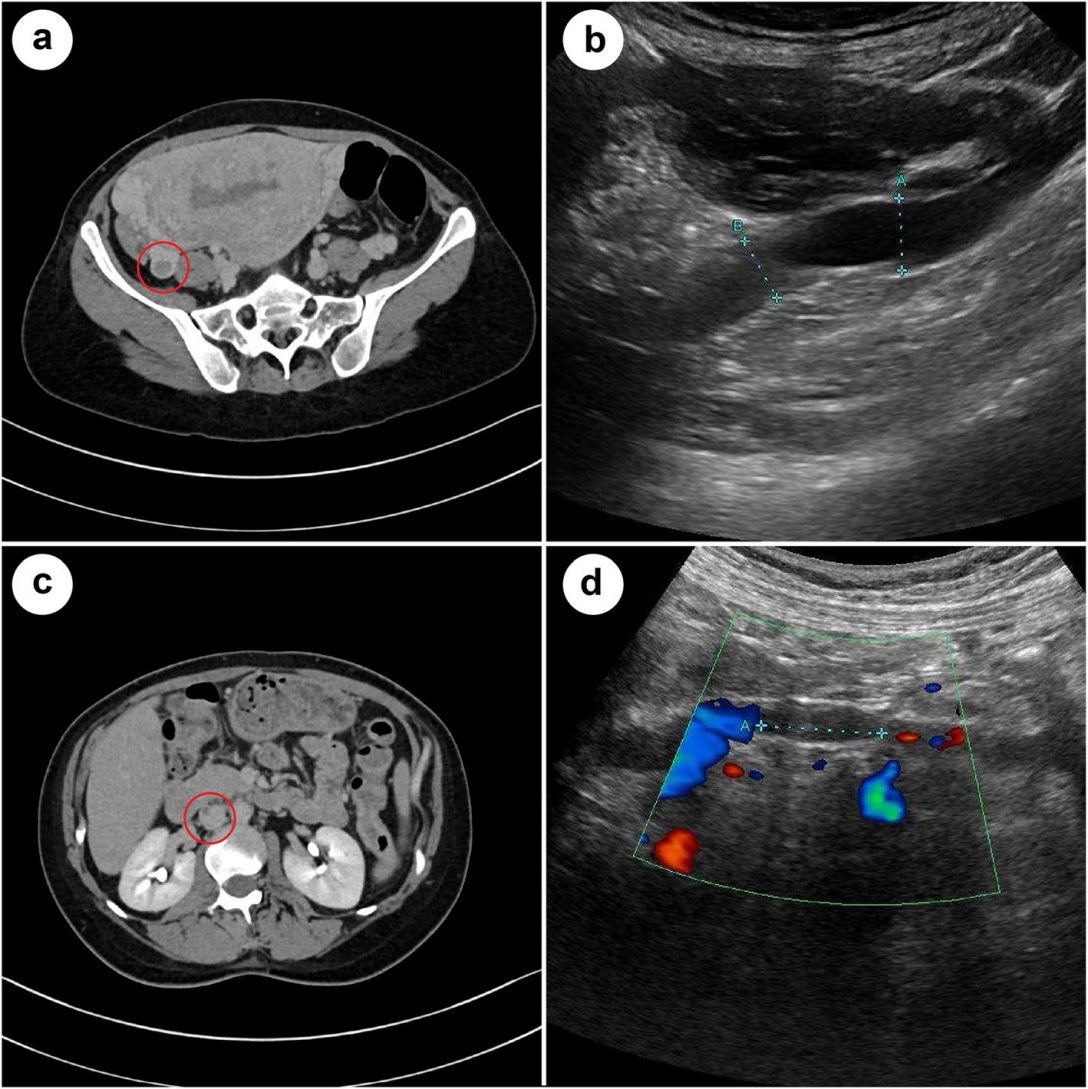
Computed tomography and trans-abdominal ultrasonography of right ovarian vein thrombosis on the days of detection (**a**, **b**) and extension to the inferior vena cava (**c**, **d**)

**Fig. 2:**
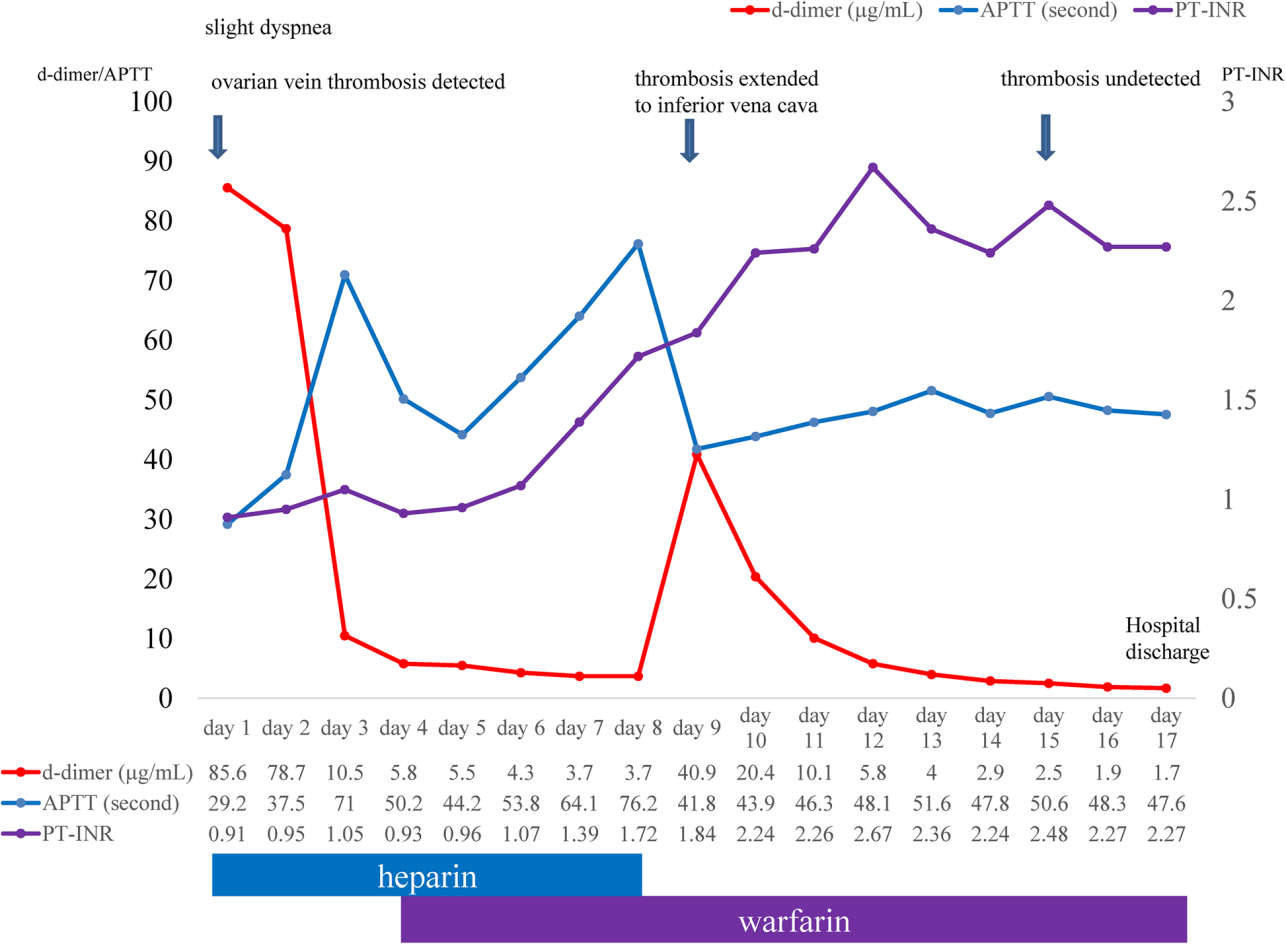
Clinical course of this case

One day after delivery, she experienced mild dyspnea whilst moving around, with percutaneous saturation of 98% (room air) and a hemoglobin level of 10.3 g/dL. The heart rate occasionally increased up to 125 beats per minute. The assessment of her legs did not show any clinical signs of DVT. Although a massive PTE was not suspected, laboratory tests were performed, which showed an elevated D-dimer level of 85.6 μg/mL. CT revealed no signs of PTE and no signs of DVT present in the legs; however, a right OVT of 12 mm (major axis diameter: 40 mm) and a dilated right ovarian vein of 15 mm were revealed (Fig. 1a). Considering the risk of PTE from OVT, heparinization (initial dose, 15,000 U/day) was performed by continuous venous infusion, reaching the appropriate range of activated partial thromboplastin time (APTT). On the next day, trans-abdominal ultrasound (TAUS) also revealed a right OVT and a dilated right ovarian vein of 14 mm (Fig. 1b); the left ovarian vein was 5 mm. Additionally, warfarin (initial dose, 3 mg/day) was administered per os on day 4 after delivery; heparin was ceased on day 8 under appropriate Prothrombin International Normalized Ratio (PT-INR) by administration of warfarin (Fig. 2).

After initiating heparin administration, D-dimer levels decreased day by day (Fig. 2). However, on day 9 after delivery, D-dimer levels suddenly increased to 40.9 μg/mL despite adequate anticoagulation treatment. CT revealed an extended thrombosis from the right ovarian vein to the IVC; a thrombosis of 7 mm in the right ovarian vein and a thrombosis of 6 mm in IVC (major axis diameter: 48 mm) were detected (Fig. 1c). Because the size of the thrombosis decreased, and the location of the thrombosis was out of the range for an IVC filter, anticoagulation treatment was continuously administered. On day 10 after delivery, TAUS also revealed an extended thrombosis from the right ovarian vein to IVC (Fig. 1d), although no symptoms were detected.

Warfarin administration was continued, and D-dimer levels decreased again (Fig. 2). The final dose of warfarin was 2 mg/day. On day 15 after delivery, TAUS showed no signs of thrombosis on the right ovarian vein and IVC. Based on the decreased D-dimer levels of 1.7 μg/mL and the absence of signs of thrombosis on TAUS, the patient was discharged from the hospital on day 17 after delivery (Fig. 2).

On day 36 after delivery, D-dimer level was 0.1 μg/mL; CT (day 37) and TAUS (day 38) showed no signs of thrombosis in the right ovarian vein and IVC. Thus, warfarin administration was ceased on day 38 after delivery. On day 59 after delivery, thrombophilia test results were as follows: antithrombin-III: 93%; protein-C activity: 148%; protein-S activity: 85%; antiphospholipid antibodies were negative. In addition, both PT-INR and APTT were within normal range and re-elevation of D-dimer was not observed, confirming no ongoing coagulation abnormality in this patient.

## Discussion and conclusions

This case demonstrates the potentially devastating outcome of a postpartum OVT that occurred after a low-risk vaginal delivery, despite the woman appearing to be asymptomatic. Anticoagulation treatment could avoid the lethal conditions due to PTE.

A state of being hypercoagulable and the risk of venous thromboembolism in pregnant women could easily cause OVT. According to a previous study, several differences in OVT characteristics were reported between women with and without pregnancies [3]; there were higher rates of causative and underlying risk factors present in pregnant women, including fever, nausea/vomiting, leukocytosis, increased lactate dehydrogenase levels, increased anticoagulation, possibility of added antibiotic medication, and longer duration of hospitalization [3]. No risk factors for postpartum venous thromboembolism, such as age over 35, scarred uterus, intrauterine infection, antiphospholipid syndrome, and emergency cesarean section [10], were reported in the case presented here, except for potentially hypovolemic condition indicated by transient tachycardia.

Symptomatic OVT is often indicated by fever and lower abdominal pain; furthermore, nonspecific symptoms, such as nausea/vomiting, anorexia, malaise, or dyspnea, can occur [11]. Moreover, septic thrombophlebitis could show symptoms such as fever and pelvic pain more frequently [11, 12]. The patient described here developed mild dyspnea associated with walking; however, its underlying etiology was not defined. A possible explanation is that a minute thrombosis not detectable by CT may have reached the pulmonary arteries from the right OVT. Other pulmonary events specific to a postpartum condition, such as transient amniotic fluid embolism, cannot be excluded and may have occurred in a short period.

Asymptomatic OVT is considered potentially benign compared to symptomatic OVT, and treatment may be deemed unnecessary [4]. However, the extension of OVT to IVC in this case showed the potential critical risks of PTE [3], and PTE may have been caused by asymptomatic OVT, as described in a previous case report [13]. Although anticoagulation therapy is indicated for patients with symptomatic postpartum OVT, conclusive recommendations for the treatment of all postpartum OVT cases have not been suggested because of the small number of studies [4]. In the present case, the patient was administered anticoagulant therapy (heparin and warfarin), and extension from OVT to PTE was avoided without any side effects. The duration of warfarin administration was determined based on reduction of D-dimer levels, disappearance of OVT on CT and TAUS, and potential side effects associated with warfarin, without definite recommendations for asymptomatic OVT treatment. Thus, the patient reported here was followed up by regular laboratory tests after cessation of warfarin administration. Although a number of case reports have demonstrated the successful management of OVT by direct oral anticoagulants (DOACs) in non-pregnant women [14, 15], warfarin rather than DOACs has been suggested for pregnant and breastfeeding women despite limited data [16, 17]. An IVC filter was not indicated because there was no contraindication to anticoagulation therapies [11]. Optimal anticoagulation treatments for both symptomatic and asymptomatic OVT should be clarified in further studies.

Previous studies have reported a physiological increase in D-dimer levels in pregnant women throughout pregnancy [18–20]. The D-dimer levels in postpartum women were also elevated, returning to pre-pregnancy levels 30–45 days after delivery [21]; the median values for D-dimer at term, day of delivery, and days 1, 3, 10, 30, and 45 after spontaneous vaginal delivery were 1.385 μg/mL, 3.641 μg/mL, 1.992 μg/mL, 1.203 μg/mL, 1.214 μg/mL, 0.331 μg/mL, and 0.241 μg/mL, respectively [21]. Moreover, a previous prospective cohort study reported that D-dimer levels over 3.70 μg/mL were significantly associated with increased incidence of postpartum venous thromboembolism [10]. In the present case, D-dimer levels until day 13 after delivery were above 3.0 μg/mL, indicating the presence of DVT (TAUS showed no signs of thrombosis on day 15 after delivery). Moreover, re-elevated D-dimer levels under appropriate PT-INR levels following warfarin administration suggested increased fibrinolysis of the existing thrombosis, which led to CT investigation and detection of an extended thrombosis.

Proper universal screening for DVT, including OVT, in pregnant women, especially during the postpartum period after vaginal delivery, has not been established in Japan. In a previous study, laboratory data were nonspecific and often within normal limits in women with OVT [3]. Although the negative predictive value of the highly sensitive D-dimer test is high, its specificity is low, even in general populations [22]. Additionally, it is conflicting whether thrombophilia testing is useful for evaluating OVT [1, 3]. In both symptomatic and asymptomatic OVT cases, nonspecific clinical findings may not lead to certain diagnoses of OVT. Therefore, an OVT diagnosis may be based on a clinical index of high suspicion due to the vague presentation of OVT. However, in some cases, higher D-dimer levels may lead to suspicion of DVT, as in this case. Furthermore, imaging tests, such as CT and ultrasound, are essential in assisting OVT diagnosis [3]; specifically, CT has a high sensitivity and specificity because of the gonadal vein that can easily be visualized [11], and TAUS is helpful due to its real-time evaluation.

In conclusion, even asymptomatic OVT may be extended to IVC and may cause critical PTE. Further studies are required to clarify the utility of D-dimer and imaging tests for the universal screening of DVT, including OVT, and optimal treatments for both symptomatic and asymptomatic OVT in pregnant and postpartum women.

## Data Availability

Data sharing is not applicable to this article as no datasets were generated or analyzed during the current study.

## Abbreviations

OVT: Ovarian vein thrombosis
PTE: Pulmonary thromboembolism
CT: Computed tomography
DVT: Deep vein thrombosis
TAUS: Trans-abdominal ultrasonography
IVC: Inferior vena cava
US: Ultrasound
PT-INR: Prothrombin international normalized ratio
APTT: Activated partial thromboplastin time
